# A Rare Case of Small Bowel Intussusception Secondary to Angiomyxoma With Concurrent Ovarian Cystadenofibroma

**DOI:** 10.7759/cureus.76634

**Published:** 2024-12-30

**Authors:** Sandeepa Dadigamuwage, Mafaiz Jaufer

**Affiliations:** 1 Colorectal Surgery, University Hospitals Plymouth NHS Trust, Plymouth, GBR

**Keywords:** adult small bowel intussusception, angiomyxoma, bilateral salpingo-oophorectomy, copes method, cystadenofibroma, general surgery, histopathology, intussusception, radiology, small bowel obstruction

## Abstract

Adult intussusception is an uncommon condition that constitutes a small percentage of cases of bowel obstruction in adults. Unlike its paediatric counterpart, it is often linked to an underlying pathology, necessitating surgical interventions for diagnosis and treatment. This report discusses a case involving a 54-year-old woman who presented with symptoms of small bowel obstruction, including abdominal pain, nausea, and constipation, along with a one-month history of weight loss. Imaging revealed small bowel intussusception and an abnormal pelvic mass. Surgical intervention confirmed an ileo-ileal intussusception with an intraluminal mass as the lead point and a concurrent ovarian mass. The histopathological analysis identified the lead point as a benign small bowel angiomyxoma alongside a benign ovarian cystadenofibroma. The patient recovered well following laparoscopic reduction of the intussusception, bowel resection, and bilateral salpingo-oophorectomy. This case highlights the diagnostic challenges posed by adult intussusception due to its nonspecific presentation and underscores the importance of imaging, surgical management, and multidisciplinary collaboration. The rare finding of a small bowel angiomyxoma as a lead point emphasises the diverse aetiology of this condition.

## Introduction

Adult small bowel intussusception is a rare but clinically significant condition, accounting for approximately 5% of all intussusception cases and 1-5% of bowel obstructions in adults. Unlike in paediatric cases, where intussusception is often idiopathic, the condition in adults is commonly associated with an underlying pathological lead point, such as a tumour, polyp, or Meckel’s diverticulum. This distinction underscores the necessity for surgical intervention in most adult cases to address both the intussusception and its underlying cause [1].

The clinical presentation of adult intussusception is often nonspecific, ranging from intermittent abdominal pain and nausea to acute intestinal obstruction. This variability frequently delays diagnosis, making imaging studies, particularly computed tomography (CT), indispensable. CT scans typically reveal the characteristic "target" or "sausage-shaped" mass, which is diagnostic of intussusception [2].

Surgical management remains the cornerstone of treatment, as most cases require resection of the affected bowel segment to address the lead point and reduce the risk of recurrence. Here, we report a rare case of adult small bowel intussusception secondary to a small bowel angiomyxoma with concurrent ovarian cystadenofibroma. This report details the diagnostic workup, surgical intervention, and clinical outcome, contributing to the growing body of literature on this condition [3].

## Case presentation

**Figure 1:**
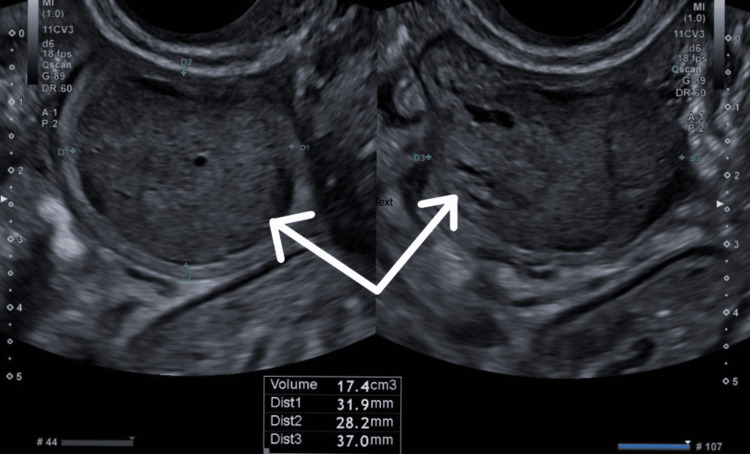
Transvaginal ultrasound imaging showing a left ovarian mass

A 54-year-old previously healthy patient was admitted to the hospital with worsening epigastric pain accompanied by nausea and vomiting. She had initially presented to the hospital five days prior with similar symptoms. During her initial visit, a gynaecological assessment revealed elevated CA 125 levels and a left ovarian mass with free fluid in the pouch Douglas, identified via transvaginal ultrasound (Figure 1). She was discharged with a plan for an urgent outpatient CT scan of the abdomen and pelvis for further evaluation.

Upon this admission, she also reported indigestion and absolute constipation lasting five days. Additionally, she described a recent one-month history of weight loss and loss of appetite. She denied any history of vaginal bleeding, and her last menstruation occurred 15 years ago. Her gynaecological history included two elective lower-segment caesarean sections, both without complications. She had no family history of gynaecological cancers, although her brother had been diagnosed with leukaemia. She reported no known drug allergies and was not on any routine medications.

On clinical examination, the patient appeared pale and cachectic, with mild tenderness in the right iliac fossa. Otherwise, her abdominal examination was unremarkable. A digital rectal examination revealed soft stools with no masses or contact bleeding. Initial haematological investigations are outlined in Table 1.

**Table 1:** Initial haematological investigation findings eGFR: estimated glomerular filtration rate, CRP: C-reactive protein, CA 125: cancer antigen 125

Parameter	Results	Reference range
eGFR	>90	ml/min/1.73m^2^
CRP	8	0.1-5mg/L
Sodium	133	133-146 mmol/L
Potassium	3.3	3.5-5.3 mmol/L
Urea	3.4	2.5-7.8 mmol/L
Creatinine	61	64-104 mmol/L
White cells	8.4	3.6-9.2 x10^9^/l
Haemoglobin	137	130-175 g/l
Platelets	393	150-450 x10^9^/l
CA 125	53	0-35 ku/L

**Figure 2:**
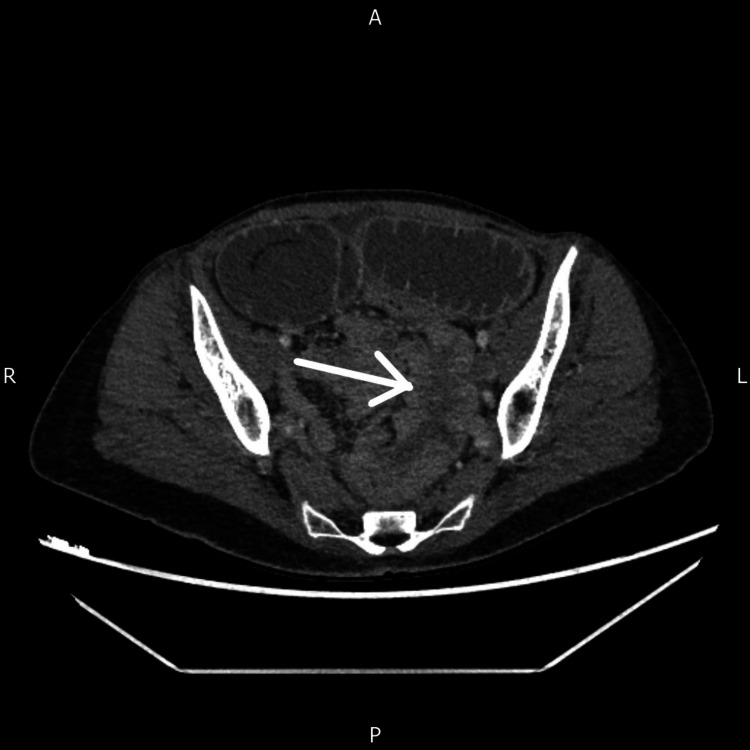
CT scan of the abdomen and pelvis showing a small bowel intussusception as observed in the axial view CT: computed tomography

An urgent inpatient CT scan of the abdomen and pelvis with contrast was performed, revealing high-grade small bowel obstruction with small bowel intussusception (Figure 2) and an abnormal pelvic mass. A multidisciplinary discussion with the gynaecology and general surgery teams concluded with the decision to proceed with diagnostic laparoscopy.

**Figure 3:**
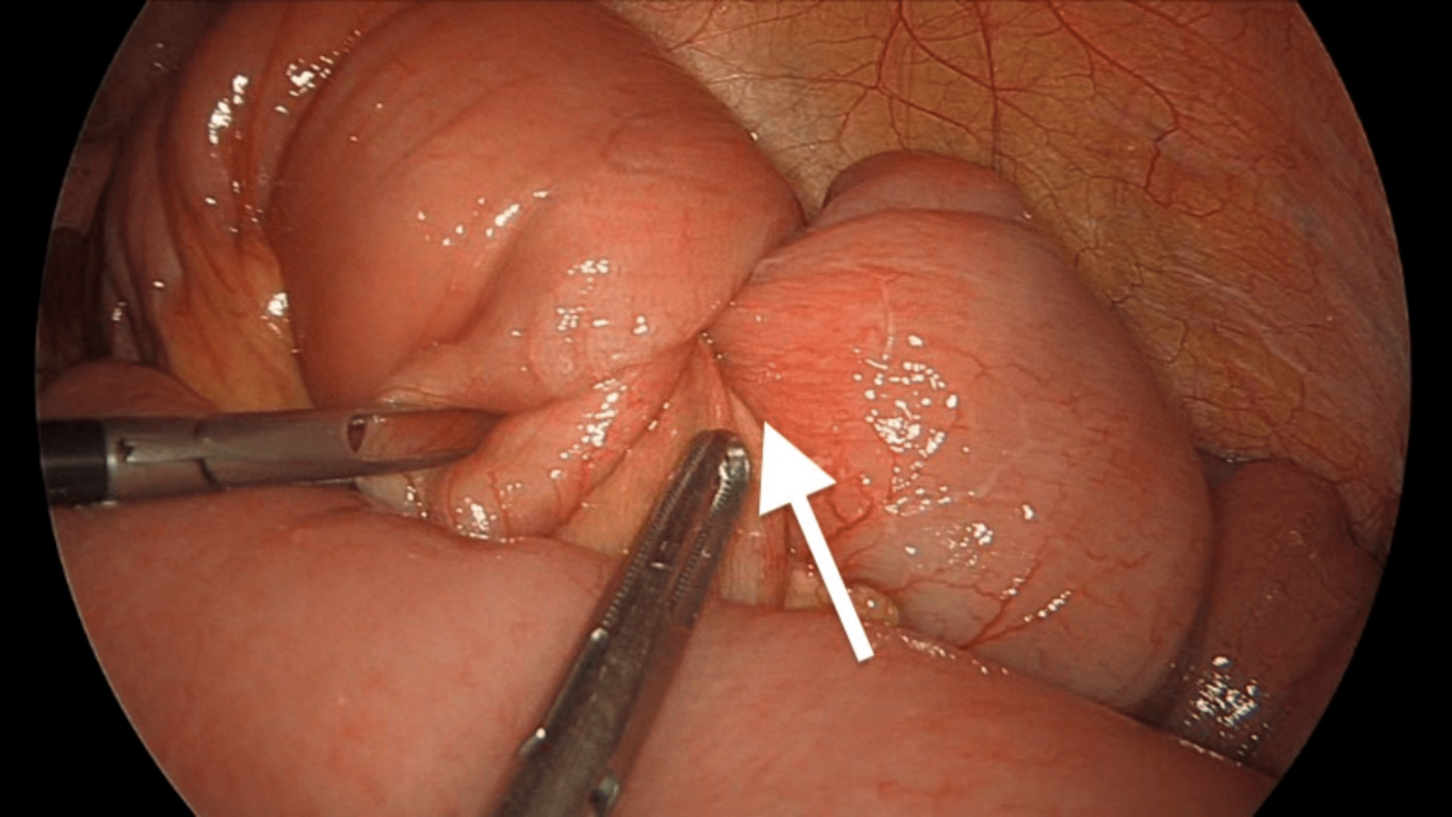
Ileo-ileal intussusception identified during diagnostic laparoscopy

**Figure 4:**
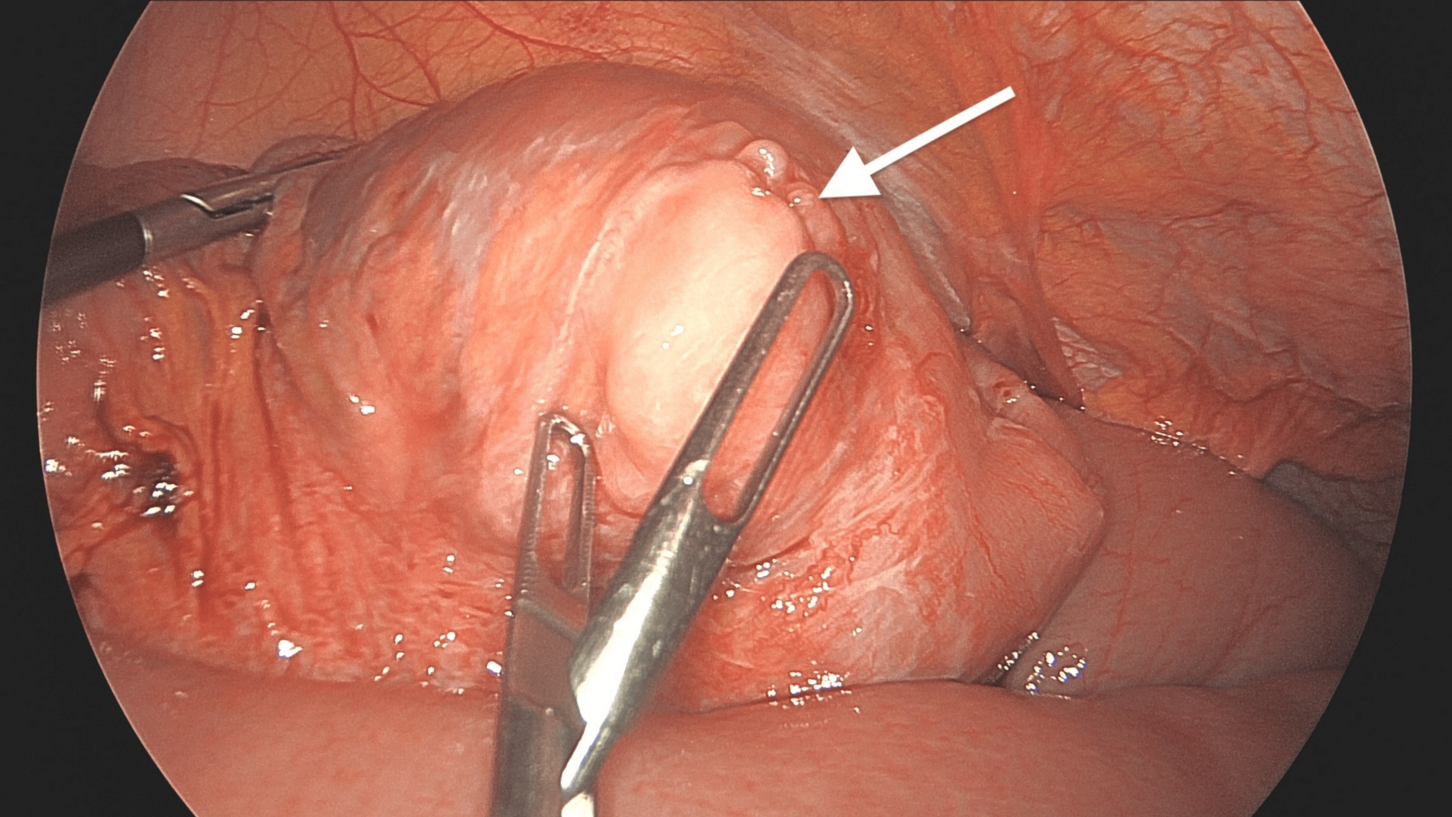
Leading point of the intussusception after reduction using Copes method

**Figure 5:**
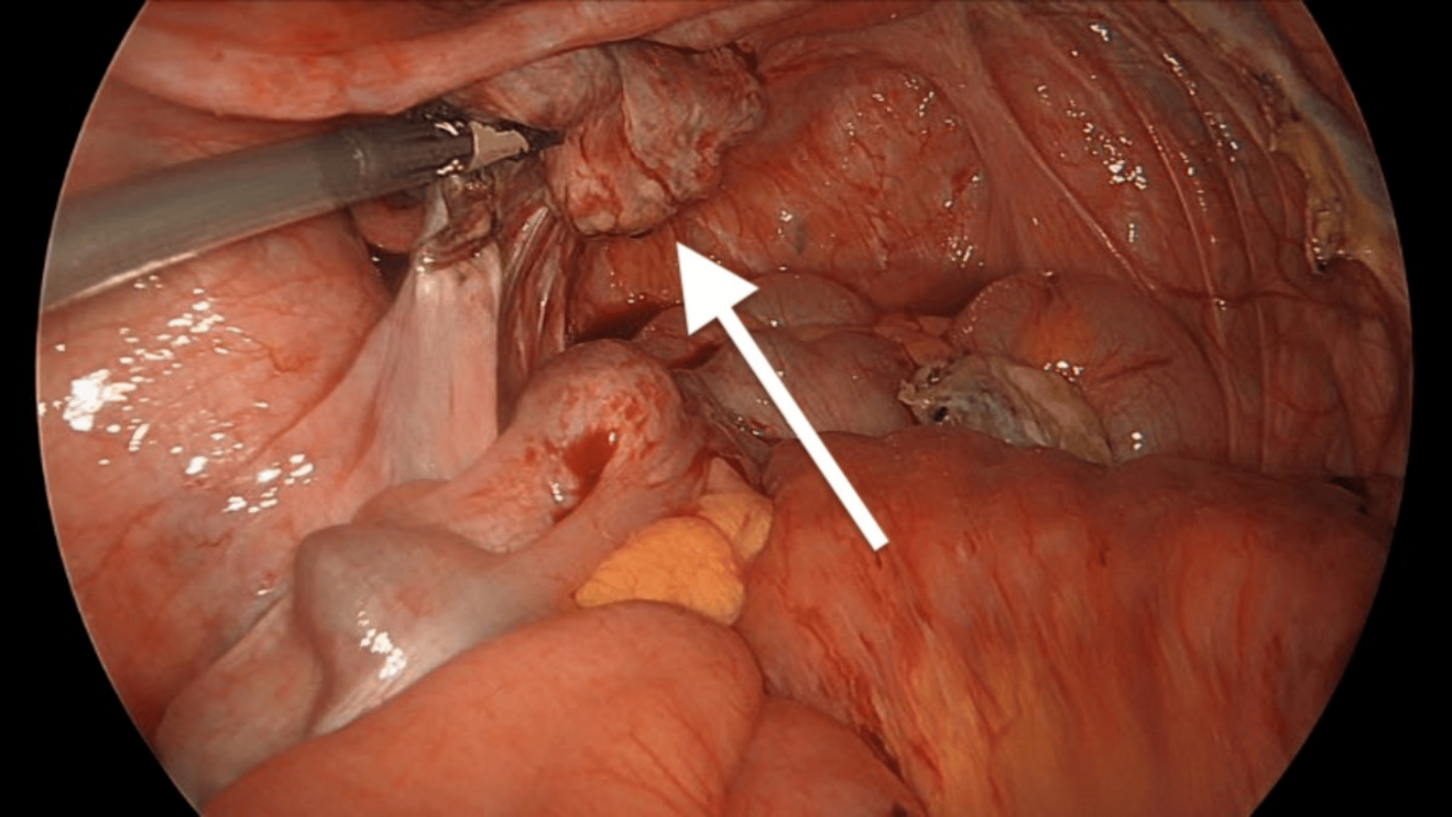
Mass arising from the left ovary after performing right salpingo-oophorectomy

The patient underwent diagnostic laparoscopy, laparoscopic small bowel resection with primary anastomosis, and bilateral salpingo-oophorectomy. Given the preoperative findings, including elevated CA 125 levels and imaging studies consistent with pathology, as well as the patient’s postmenopausal status, a decision was made to proceed with small bowel resection and bilateral salpingo-oophorectomy to address both the intussusception and the ovarian pathology. During surgery, an ileo-ileal intussusception was identified (Figure 3), with the lead point being an intraluminal mass (Figure 4). The intussusception was reduced using the laparoscopic Copes method, and the affected part of the small bowel was resected. Additionally, intra-abdominal free fluid was observed, and a mass arising from the left ovary was noted (Figure 5); however, there were no features suggestive of disseminated disease. The surgical specimens and intra-abdominal free fluid samples were sent for histological analysis. The patient recovered uneventfully during the postoperative period.

**Figure 6:**
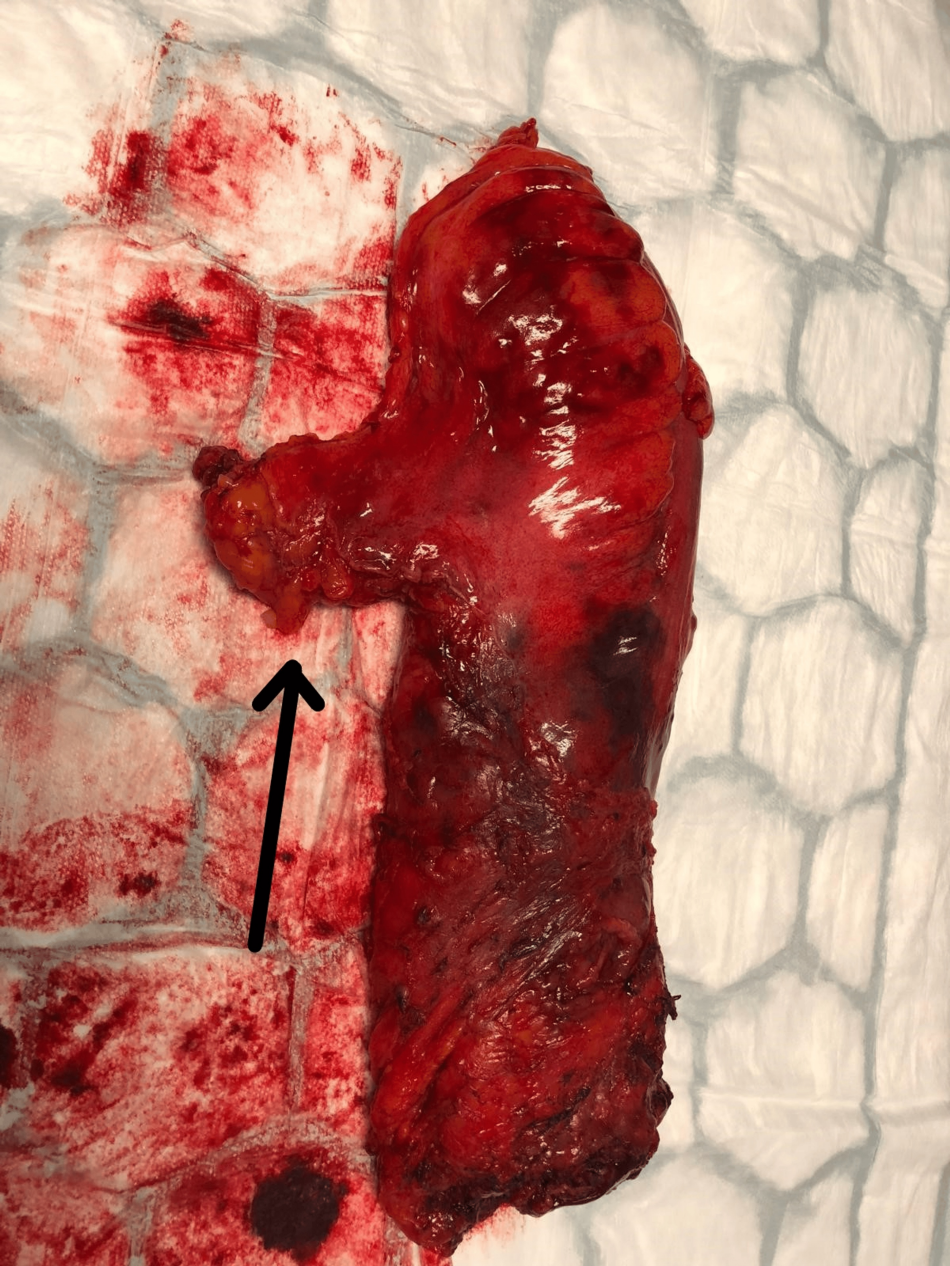
Resected specimen of the small bowel demonstrating a pathological lead point associated with intussusception

Postoperative analysis of the intra-abdominal free fluid revealed sheets of benign reactive mesothelial cells and mixed inflammatory cells. Histological examination of the specimens showed a morphologically normal right ovary and the left ovary containing a large benign cystadenofibroma measuring 35 mm × 15 mm × 12 mm. The small bowel specimen revealed a benign angiomyxoma measuring 55 mm × 40 mm × 40 mm (Figure 6). The patient recovered uneventfully and was discharged on the fourth postoperative day.

## Discussion

Adult intussusception is a rare but important clinical entity with a significantly different aetiology, presentation, and management compared to its paediatric counterpart. The underlying pathological lead point in adult intussusception, often a benign or malignant tumour, distinguishes it from the idiopathic nature observed in most paediatric cases [4]. In this report, the patient’s intussusception was caused by a benign angiomyxoma, a highly unusual aetiology, highlighting the diverse pathology that can precipitate this condition.

The clinical presentation of adult intussusception is highly variable, frequently delaying diagnosis. In this case, the patient presented with progressive abdominal pain, nausea, vomiting, and absolute constipation, features that align with the most common symptoms reported in the literature [5]. However, the non-specificity of these symptoms often leads to diagnostic ambiguity. Imaging studies, particularly contrast-enhanced CT, play a pivotal role in the timely diagnosis of adult intussusception. CT findings typically include the “target” or “sausage-shaped” mass indicative of bowel telescoping, as observed in this patient [6]. These findings were instrumental in directing the surgical approach and multidisciplinary team decision-making.

Surgical management is considered the gold standard for adult intussusception due to the high likelihood of an underlying pathological lead point. The decision to perform a small bowel resection with primary anastomosis in this case aligns with established best practices, ensuring both the resolution of the obstruction and histological examination of the lead point [7]. The additional discovery of an ovarian mass and intra-abdominal free fluid necessitated a broader surgical intervention, including bilateral salpingo-oophorectomy, to address potential concurrent pathology. The absence of disseminated disease in this patient was a favourable finding, as small bowel intussusception secondary to malignancy has been associated with a poorer prognosis [4].

Histological examination confirmed the rare diagnosis of benign angiomyxoma as the lead point of intussusception. Angiomyxomas are mesenchymal tumours that rarely occur within the gastrointestinal tract [6]. While benign, they can act as mechanical obstructions when located intraluminally, as in this case. Additionally, the finding of a benign cystadenofibroma in the ovary underscores the importance of a thorough histopathological evaluation in cases involving multiple pathologies.

This case emphasises the importance of a multidisciplinary approach in managing complex presentations of adult intussusception. Collaboration between surgical and gynaecological teams facilitated timely intervention and comprehensive treatment, optimising patient outcomes. The postoperative recovery of this patient was uneventful, reflecting the efficacy of the chosen surgical approach and the benign nature of the pathology.

## Conclusions

This case highlights the diagnostic and therapeutic challenges of adult intussusception. The combination of a rare lead point and concurrent gynaecological pathology demonstrates the need for vigilance and a systematic approach to managing such cases. While imaging studies are indispensable for diagnosis, surgical intervention remains critical for both treatment and definitive diagnosis. This report contributes to the growing body of evidence on the varied presentations and management of adult intussusception, emphasising the role of multidisciplinary care and individualised surgical planning.
